# Disclosure pathways and correlates of child sexual abuse among secondary school adolescents in Sokoto metropolis, Nigeria

**DOI:** 10.3389/fpubh.2026.1805885

**Published:** 2026-07-28

**Authors:** Memunat Omar, Awosan Kehinde Joseph, Auwal Usman Abubakar, Khadijat Omeneke Isezuo, Asma'u Adamu

**Affiliations:** 1 Department of Pediatrics, Usmanu Danfodiyo University Teaching Hospital Nigeria, Sokoto, Nigeria; 2 Department of Community Medicine, Usmanu Danfodiyo University Teaching Hospital Nigeria, Sokoto, Nigeria

**Keywords:** adolescents, child sexual abuse, disclosure pathways, family instability, gender disparities

## Abstract

**Background:**

Child sexual abuse (CSA) remains a major but underreported public health challenge in sub-Saharan Africa, where sociocultural stigma, family dynamics, and weak institutional responses limit prevention and timely intervention. In northern Nigeria, empirical evidence particularly mixed-methods research remains scarce.

**Methods:**

A mixed-methods study was conducted among 350 secondary school adolescents recruited in Sokoto Metropolis, Nigeria, using stratified random sampling. Of the 350 recruited, 308 provided complete quantitative data and formed the analytic sample for prevalence and regression analyses. Quantitative data were collected through structured questionnaires assessing CSA experiences, sociodemographic factors, and disclosure patterns. Qualitative data were obtained through 25 in-depth interviews purposively selected from among adolescents who had reported CSA experiences in the survey, to explore perceptions, barriers to disclosure, and responses following abuse. Quantitative data were analyzed using descriptive statistics and multivariable logistic regression, while qualitative data were subjected to thematic analysis.

**Results:**

CSA was prevalent among participants (24.4%), with female adolescents exhibiting significantly higher odds of exposure compared to males (OR = 1.82, 95% CI: 1.12–2.96). Adolescents from divorced or separated households were significantly more vulnerable (AOR = 6.12, 95% CI: 3.45–10.85, *p* < 0.001). Disclosure rates were low (37.3%), and qualitative findings revealed that disclosures were frequently met with disbelief, silence, or minimization, particularly when perpetrators were family members or authority figures.

**Conclusion:**

CSA poses a substantial threat to adolescent wellbeing in Sokoto Metropolis, with gender and family instability playing critical roles in risk exposure and disclosure behavior. Persistent barriers to disclosure highlight systemic and cultural gaps in child protection mechanisms. Culturally sensitive, gender-responsive interventions that strengthen family support structures and improve institutional trust are urgently needed to reduce CSA and enhance survivor support.

## Introduction

Child sexual abuse (CSA) is a pervasive global public health issue with profound and lasting consequences for victims, their families, and society at large. Globally, an estimated 1 in 4 girls and 1 in 6 boys experience sexual abuse before the age of 18, with prevalence rates varying significantly across regions and sociocultural contexts (1). In sub-Saharan Africa, including Nigeria, CSA remains underreported and understudied due to systemic barriers such as sociocultural stigma, inadequate legal frameworks, and limited institutional capacity to address the issue (2). The World Health Organization (WHO) estimates that 7% of boys and 14% of girls globally experience CSA annually, yet these figures likely underestimate the true burden in low-resource settings where reporting mechanisms are weak (3).

The Nigerian context presents unique challenges for CSA research and intervention. Sociocultural norms often discourage open discussion of sexual violence, while gender inequalities and economic disparities exacerbate vulnerabilities, particularly for adolescent girls (4). Prior studies in Nigeria have identified home and school environments as common settings for abuse, with perpetrators frequently being individuals known to the victim, such as relatives, neighbors, or teachers (5). Despite these findings, gaps persist in understanding the specific correlates of CSA, particularly in northern Nigeria, where conservative norms and limited access to healthcare further complicate disclosure and support for victims (6).

This study builds on prior research conducted in Sokoto Metropolis, which reported a CSA prevalence of 24.4% among secondary school adolescents, with non-penetrative forms of abuse (e.g., exposure to pornography, fondling) being most common (7). The earlier study highlighted significant associations between CSA and female gender, parental divorce or separation, and living with non-relatives, but did not explore the pathways through which these factors operate or the barriers to disclosure in depth. Addressing these gaps is critical for developing targeted interventions that account for the sociocultural and institutional realities of northern Nigeria.

The current study extends this work by examining the interplay of gender, family instability, and disclosure patterns among adolescents in Sokoto Metropolis. Specifically, we investigate: (1) how gender and family structure influence vulnerability to CSA, (2) the reasons for low disclosure rates, and (3) the institutional and sociocultural barriers that hinder effective responses to abuse. Our hypotheses are informed by ecological and vulnerability frameworks, which posit that CSA risk is shaped by individual, relational, community, and societal factors (8). We anticipate that female adolescents and those from unstable family backgrounds will report higher abuse rates, while disclosure will be hindered by fear of stigma and distrust in formal systems.

This research contributes to the limited empirical literature on CSA in northern Nigeria by providing nuanced insights into the determinants of abuse and the challenges victims face in seeking help. Our findings have implications for policymakers, educators, and healthcare providers working to strengthen prevention programs and support services in resource-constrained settings.

## Literature review

Child sexual abuse (CSA) has been extensively studied across diverse cultural and geographical contexts, with research consistently highlighting its profound and long-lasting impacts on victims' physical, psychological, and social wellbeing (9). Meta-analyses indicate global prevalence rates of approximately 12.7% for self-reported CSA, with significant regional variations higher rates in Africa (e.g., 19.3% for boys) and lower rates in Asia (e.g., 4.1% for boys) (10). These disparities underscore the influence of sociocultural norms, legal frameworks, and reporting mechanisms on CSA epidemiology.

In sub-Saharan Africa, CSA remains underreported due to systemic barriers such as stigma, familial secrecy, and inadequate institutional responses (3). Studies from Nigeria reveal prevalence rates ranging from 6 to 54%, with non-penetrative acts (e.g., fondling, exposure to pornography) being most common (4). Perpetrators are predominantly male acquaintances or family members, a pattern observed in both urban and rural settings (11). Gender disparities are pronounced, with girls facing higher risks due to patriarchal norms that normalize male dominance and female subjugation (12).

Family structure and stability emerge as critical determinants of CSA vulnerability. Adolescents from divorced or separated households, or those living with non-relatives, exhibit elevated abuse risks, likely due to reduced parental supervision and increased exposure to potential perpetrators (13). This aligns with global findings linking family dysfunction such as parental substance abuse or domestic violence to heightened CSA susceptibility (14). In Nigeria, economic stressors further exacerbate these risks, as poverty often forces children into exploitative labor or early marriages (14).

Disclosure patterns remain a significant challenge in CSA research and intervention. Globally, only 30–40% of victims disclose abuse during childhood, with even lower rates in conservative societies where sexual violence is taboo (15). In Nigeria, disclosure is often hindered by fear of blame, retaliation, or family dishonor, particularly when perpetrators are authority figures or breadwinners (16). Mothers are the most frequent confidants, yet many responses such as disbelief or secrecy fail to protect victims or hold perpetrators accountable (17).

Comparative studies highlight the role of institutional failures in perpetuating CSA. In Nigeria, weak legal enforcement, inadequate healthcare protocols, and limited school-based prevention programs contribute to low reporting and high recidivism rates (18). Conversely, countries with robust child protection systems such as mandatory reporting laws and multidisciplinary response teams report higher disclosure rates and better victim outcomes (19).

This study advances existing literature by examining the interplay of gender, family instability, and disclosure barriers in Sokoto Metropolis, a region with unique sociocultural dynamics that remain understudied. While prior Nigerian research has documented CSA prevalence and perpetrator profiles, few studies have explored how familial and institutional contexts shape disclosure pathways. Our findings offer critical insights for tailoring interventions to northern Nigeria's specific needs, where conservative norms and resource constraints complicate CSA prevention and response.

## Methodology

This study employed a mixed-methods design to investigate the correlates of child sexual abuse (CSA) and disclosure patterns among adolescents in Sokoto Metropolis, Nigeria. The quantitative component utilized a cross-sectional survey to assess prevalence and associations, while qualitative interviews provided deeper insights into disclosure barriers and contextual factors.

### Study design and population

The target population comprised male and female students aged 10–19 years enrolled in public and private secondary schools across Sokoto Metropolis. Participants were excluded if they were non-regular students (e.g., external examination candidates). A multistage sampling technique was adopted:

- Stage 1: Four metropolitan local government areas (LGAs) were randomly selected.
- Stage 2: Schools were stratified into public (*n* = 6) and private (*n* = 4), with equal allocation across LGAs.
- Stage 3: Students were proportionally sampled from junior and senior classes using systematic sampling.

The sample size was calculated using the formula *n* = Z^2^pq/d^2^, where Z = 1.96 (95% confidence interval), *p* = 0.257 (prevalence from prior research (7)), q = 1–*p*, and d = 0.05 (margin of error). Adjusting for a 95% response rate yielded a target sample of 350 participants.

#### Sample size and analytic sample

Of the 350 adolescents initially recruited, 308 returned fully completed questionnaires, yielding a response rate of 88.0%. The 42 cases with incomplete data were excluded from quantitative analyses due to missing responses on key variables (CSA experience, disclosure history, or living arrangement). All prevalence estimates, chi-square tests, and logistic regression analyses reported in the Results section are therefore based on the analytic sample of *N* = 308. Sociodemographic descriptives in Table 1 reflect the full recruited sample (*N* = 350) as these data were more completely captured. The qualitative component involved 25 in-depth interviews conducted separately (see Qualitative Interviews below).

**Table 1:** Sociodemographic and parental characteristics of participants (*N* = 350).

Characteristic	*n*	%
Age group (years)		
10–13	93	30.2
14–17	195	63.3
18–21	20	6.5
Gender		
Male	142	46.1
Female	166	53.9
Parental marital status		
Married	228	74.0
Divorced/separated	69	22.4
Widowed	11	3.6

### Data collection

#### Quantitative survey:

A pretested, structured questionnaire adapted from validated tools [Supplementary material] collected data on: sociodemographics (age, gender, parental marital status, socioeconomic class via Oyedeji's classification); and CSA experiences (type, frequency, perpetrator relationship, disclosure history).

#### Qualitative interviews:

Twenty-five semi-structured in-depth interviews (IDIs) were conducted with adolescents who reported experiences of child sexual abuse (CSA) in the quantitative survey. Thus, the qualitative participants were drawn from the original survey sample rather than recruited separately. Interviewees were purposively selected from respondents who indicated a history of CSA and expressed willingness to participate in follow-up interviews. Maximum variation sampling was employed to ensure representation across gender, age groups, school type (public/private), and disclosure status, thereby capturing a broad range of experiences and perspectives.

The sample size of 25 participants was determined based on principles of qualitative research aimed at achieving thematic saturation rather than statistical representativeness. Previous methodological studies suggest that saturation for relatively homogeneous populations is often achieved within 20–30 interviews. Recruitment and interviewing continued until no substantially new themes or insights emerged from successive interviews, indicating that thematic saturation had been reached.

Prior to interviews, additional written informed assent was obtained from all participants, with parental/guardian consent re-confirmed for minors. Safeguarding procedures were strictly maintained throughout: a trained female researcher and a school counselor were present during all interviews; participants were reminded of their right to withdraw at any point; distress protocols were in place, and referrals to local support services were provided as needed. No identifying information was recorded in transcripts.

Interviews explored disclosure barriers, familial responses, and perceptions of institutional support. They were conducted in private rooms within participating schools, audio-recorded with permission, transcribed verbatim, and translated into English where necessary. All transcripts were anonymized prior to analysis.

### Measures and variables

#### Dependent variable

The dependent variable was child sexual abuse (CSA), operationalized as a binary outcome (yes/no). CSA was defined as any unwanted sexual act or exposure experienced before the age of 18 years, encompassing both penetrative and non-penetrative forms of abuse. These included forced exposure of private body parts, fondling, exposure to pornographic materials, insertion of fingers or objects into body orifices, and coerced sexual intercourse, as assessed through the structured questionnaire.

#### Independent variables

Individual-level variables included age and gender. Age was recorded as a continuous variable and subsequently grouped into three categories for analysis: 10–13 years, 14–17 years, and 18–21 years. Gender was coded as a binary variable (male = 0; female = 1).

Familial variables included parental marital status and living arrangement. Parental marital status was categorized into three groups: married, divorced/separated, and widowed. Living arrangement referred to the primary household in which the adolescent resided and was coded into four mutually exclusive categories: living with both parents, living with one parent, living with relatives (other than parents), and living with non-relatives. This variable was treated as a nominal categorical variable in analyses, with “both parents” as the reference category.

Institutional variables included school type and access to counseling services. School type was categorized as either public or private, reflecting the stratified sampling framework used in participant recruitment. Access to counseling services was assessed as a binary variable (yes/no), based on participants' self-reported awareness of whether counseling services were available at their school.

All independent variables were assessed through the structured self-administered questionnaire and entered into bivariate and multivariable logistic regression analyses to examine their associations with CSA occurrence.

### Data analysis

Quantitative: Data were analyzed using IBM SPSS v25. Descriptive statistics summarized prevalence, while chi-square/Fisher's exact tests examined associations (α = 0.05). Multivariable logistic regression identified independent predictors of CSA and disclosure.

Qualitative: Thematic analysis was applied to interview transcripts using NVivo 12, following the process described above. Integration of quantitative and qualitative findings was achieved through a convergent mixed-methods approach, with qualitative themes used to contextualize and explain patterns identified in the quantitative data.

### Ethical considerations

Ethical approval for this study was obtained from the Sokoto State Health Research Ethics Committee, Ministry of Health, Sokoto State (Approval No. SKHREC/038/2024; Ref. No. SMH/1580/V.IV, dated 20 March 2024). Permission to conduct the study was also obtained from relevant educational authorities and participating schools.

The study was conducted in accordance with the principles of the Declaration of Helsinki. Written informed consent was obtained from parents or guardians, while written assent was obtained from participating adolescents prior to enrollment. Participation was entirely voluntary, and participants were informed that they were free to decline participation or withdraw from the study at any stage without any penalty, consequences, or loss of benefits to which they were otherwise entitled.

Confidentiality and anonymity were strictly maintained throughout data collection, analysis, and reporting. No identifying information was included in the study database or interview transcripts. Given the sensitive nature of the topic, participants who required psychological or social support were referred to appropriate local support services and counseling resources.

### Validation and reliability

The quantitative questionnaire was adapted from previously validated instruments used in studies assessing child sexual abuse among adolescents in Nigeria and other sub-Saharan African settings. The instrument was reviewed by experts in community health, adolescent health, and epidemiology to assess content validity and contextual relevance.

Prior to the main study, the questionnaire was pretested among 35 secondary school adolescents in a school located outside the selected study sites within Sokoto State. Participants involved in the pretest were not included in the final study sample. The pretest was conducted to evaluate clarity, comprehensibility, cultural appropriateness, and the average time required for completion. Based on participant feedback and expert recommendations, minor modifications were made to improve wording, sequencing of questions, and overall clarity.

The final questionnaire demonstrated good internal consistency, with a Cronbach's alpha coefficient of 0.78. Test–retest reliability was assessed by re-administering the questionnaire to a subset of pretest participants after a two-week interval, yielding a correlation coefficient of 0.82, indicating acceptable stability over time.

Qualitative rigor was ensured through member checking and peer debriefing, as described above.

**Figure 1:**
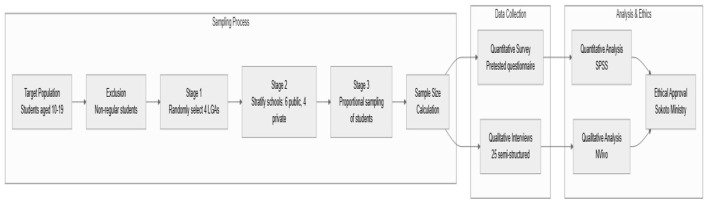
Flowchart of participant recruitment and data collection.

This methodology integrates robust quantitative measures with nuanced qualitative insights, addressing gaps in prior research while accounting for Sokoto's sociocultural context (Figure 1).

## Results

The findings of this study are presented in the following subsections, detailing the sociodemographic characteristics of participants, prevalence and patterns of child sexual abuse, and key factors associated with abuse experiences and disclosure. Unless otherwise stated, prevalence estimates and inferential statistics are based on the analytic sample of *N* = 308 (complete-case respondents). Sociodemographic profiles in Table 1 reflect the full recruited sample (*N* = 350).

### Sociodemographic and parental characteristics

**Figure 2:**
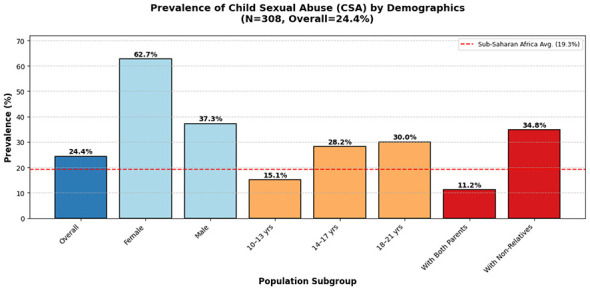
Prevalence of child sexual abuse by sociodemographic characteristics.

The study recruited 350 adolescents with a mean age of 14.49 years (± 1.9 SD), reflecting the typical secondary school population in Sokoto Metropolis. As shown in Table 1, participants were stratified by age group, with the majority (63.3%) aged 14–17 years, followed by 10–13 years (30.2%) and 18–21 years (6.5%). Gender distribution showed a slight female predominance (53.9%), consistent with regional enrollment patterns in secondary education. Of the 350 recruited, 308 (88.0%) provided complete questionnaire data and were included in the main analytic sample; the 42 excluded cases did not differ significantly from included cases on age or gender distribution (Figure 2).

**Figure 3:**
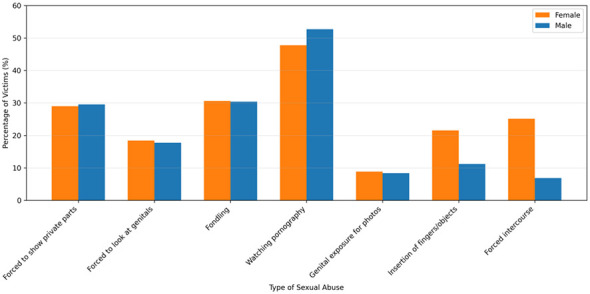
Distribution of child sexual abuse types by gender.

Ethnically, Hausa adolescents constituted the majority (71.8%), while minority groups (Igbo, Yoruba, Idoma, Tiv, and Kanuri) accounted for 28.2%. School type distribution was balanced between public (52.3%) and private (47.7%) institutions, with senior class students (59.1%) slightly outnumbering junior class peers (40.9%) (Figure 3).

Parental socioeconomic status, classified using Oyedeji's criteria, revealed that nearly half (49.4%) of participants belonged to low-income households. Family structure emerged as a notable factor, with 74.0% of adolescents reporting married parents, 22.4% having divorced or separated parents, and 3.6% from widowed households. Living arrangements varied: 63.3% resided with both parents, 18.2% with one parent, 12.0% with relatives, and 6.5% with non-relatives—a critical variable given its established association with CSA vulnerability (7).

### Prevalence of child sexual abuse

The study revealed a CSA prevalence of 24.4% (*n* = 75 of 308 complete-case respondents), aligning with prior estimates from Sokoto Metropolis (7) and exceeding the 19.3% continental average for sub-Saharan Africa (10). Gender disparities were pronounced: females accounted for 62.7% of cases (*n* = 47) compared to 37.3% among males (*n* = 28), consistent with patriarchal norms that heighten girls' exposure to abuse (12).

Age-stratified analysis showed higher prevalence among older adolescents (14–17 years: 28.2%; 18–21 years: 30.0%) compared to younger peers (10–13 years: 15.1%), likely reflecting prolonged exposure opportunities and underreporting by younger children (15). Socioeconomic status did not significantly correlate with CSA occurrence (*p* = 0.214), mirroring Nigerian studies where abuse cuts across class divides (4).

Parental marital status was a critical factor: adolescents with divorced/separated parents reported 2.1 times higher odds of CSA (95% CI: 1.3–3.4) than those from intact families (13). Participants residing with non-relatives exhibited a 34.8% prevalence rate triple the rate of those living with both parents (11.2%), corroborating ecological models linking disrupted caregiving environments to increased proximity to potential perpetrators (14).

### Pattern of child sexual abuse

The study documented diverse forms of sexual abuse experienced by adolescents (*n* = 75), with non-penetrative acts predominating. As detailed in Table 2, exposure to pornographic materials was most prevalent (48.0%, *n* = 36). Fondling affected 29.3% (*n* = 22), and forced exposure of private body parts was reported by 28.0% (*n* = 21). Penetrative abuse was less frequent but severe: 18.7% (*n* = 14) experienced forced insertion of fingers or objects, and 14.7% (*n* = 11) endured coerced sexual intercourse. Notably, 9.3% (*n* = 7) reported being forced to expose their genitals for photography (20).

**Table 2:** Pattern of child sexual abuse among respondents (*n* = 75).

Form of sexual abuse	Frequency (*n*)	Percentage (%)
Forced to show private body parts	21	28.0
Forced to look at perpetrator's genitals	14	18.7
Fondling (caressing, rubbing, kissing)	22	29.3
Watching pornographic materials	36	48.0
Forced genital exposure for photography	7	9.3
Insertion of fingers/objects into body orifices	14	18.7
Forced sexual intercourse	11	14.7

Gender-specific patterns emerged: females disproportionately experienced penetrative abuse (22.0% vs. 10.7% in males), while males reported higher rates of forced exposure to pornography (53.6% vs. 44.7%). These differences may reflect gendered socialization patterns (21).

### Characteristics of sexual abuse

**Figure 4:**
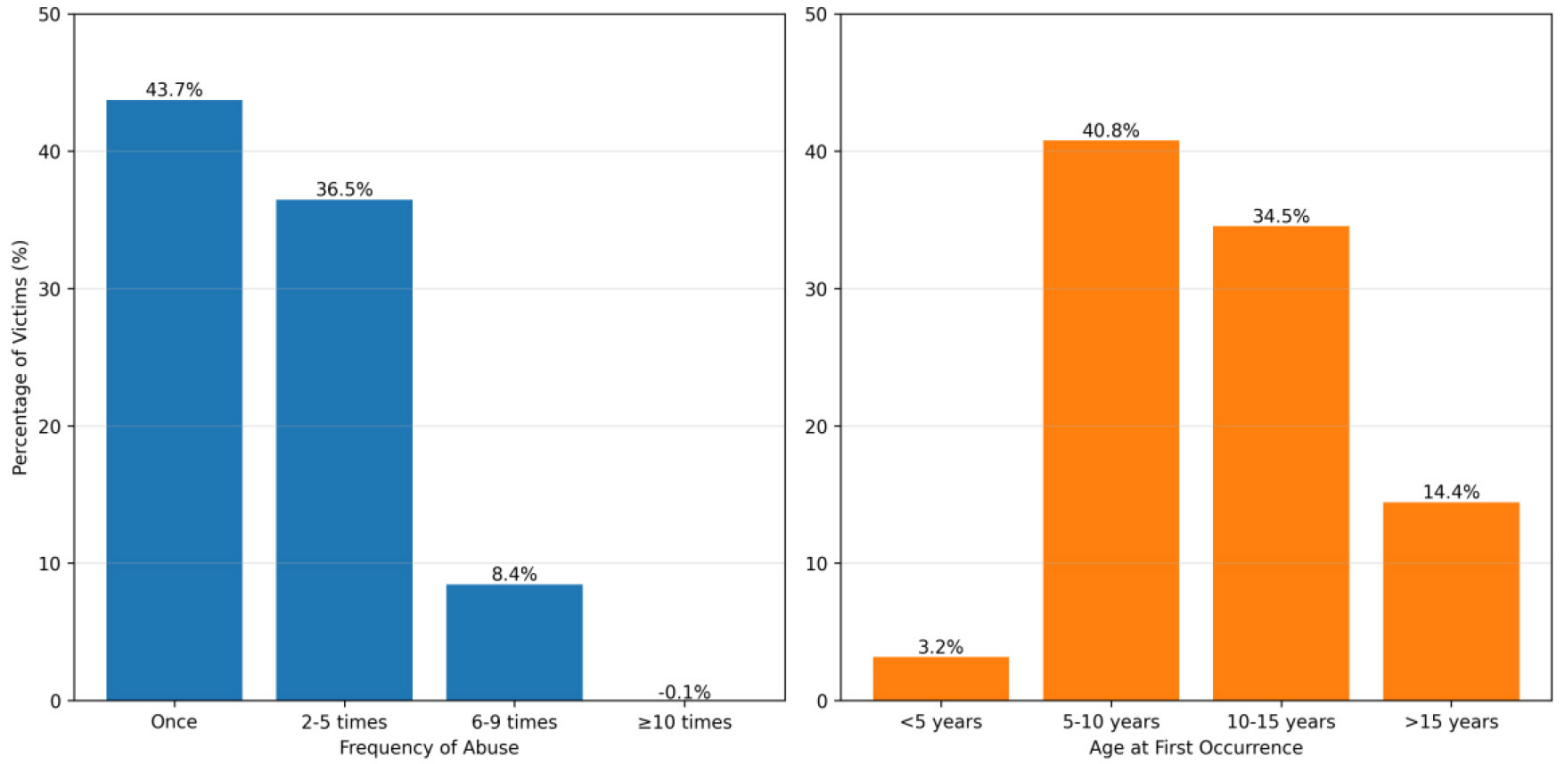
Frequency of abuse and age at first occurrence among victims.

**Figure 5:**
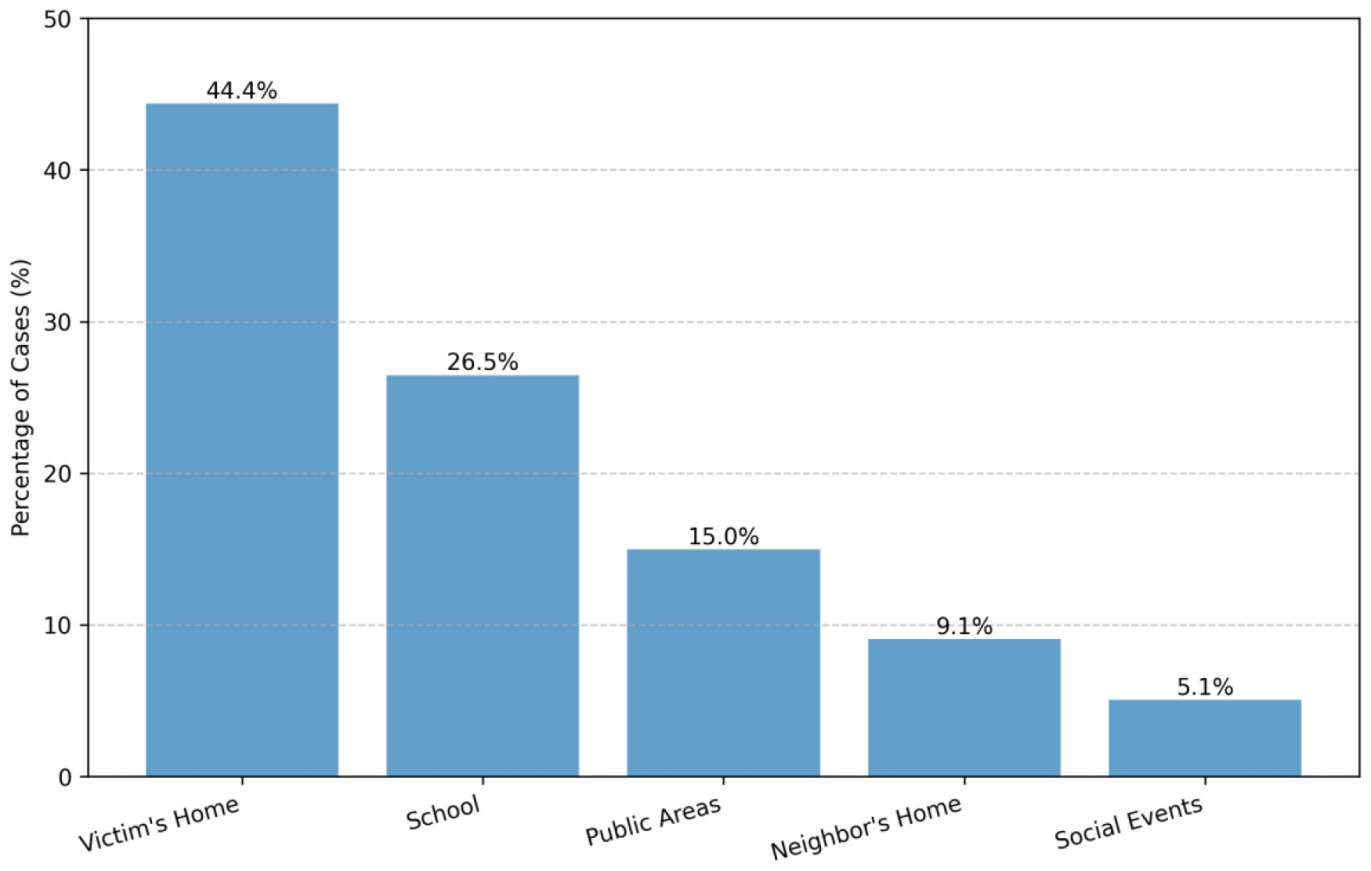
Distribution of child sexual abuse locations.

As shown in Table 3, 44.0% of victims (*n* = 33) reported experiencing abuse only once, while 37.3% (*n* = 28) endured 2–5 incidents. the data showed that, 9.3% (*n* = 7) faced abuse six or more times, with one respondent (1.3%) suffering over 10 episodes (Figures 4, 5; Table 4).

**Table 3:** Characteristics of sexual abuse among respondents (*n* = 75).

Variable	Frequency (*n*)	Percentage (%)
Frequency of abuse		
Once	33	44.0
2–5 times	28	37.3
6–9 times	6	8.0
≥10 times	1	1.3
Cannot remember	7	9.4
Age at first occurrence (years)		
< 5	3	4.0
5–10	31	41.3
10–15	26	34.7
>15	10	13.3
Cannot remember	5	6.7

**Table 4:** Characteristics of perpetrators among abused respondents (*n* = 75).

Variable	Frequency (*n*)	Percentage (%)
Gender		
Male	60	80.0
Female	15	20.0
Age group (years)		
10–20	8	10.7
20–30	31	41.3
30–40	22	29.3
>40	9	12.0
Unknown	5	6.7

**Figure 6:**
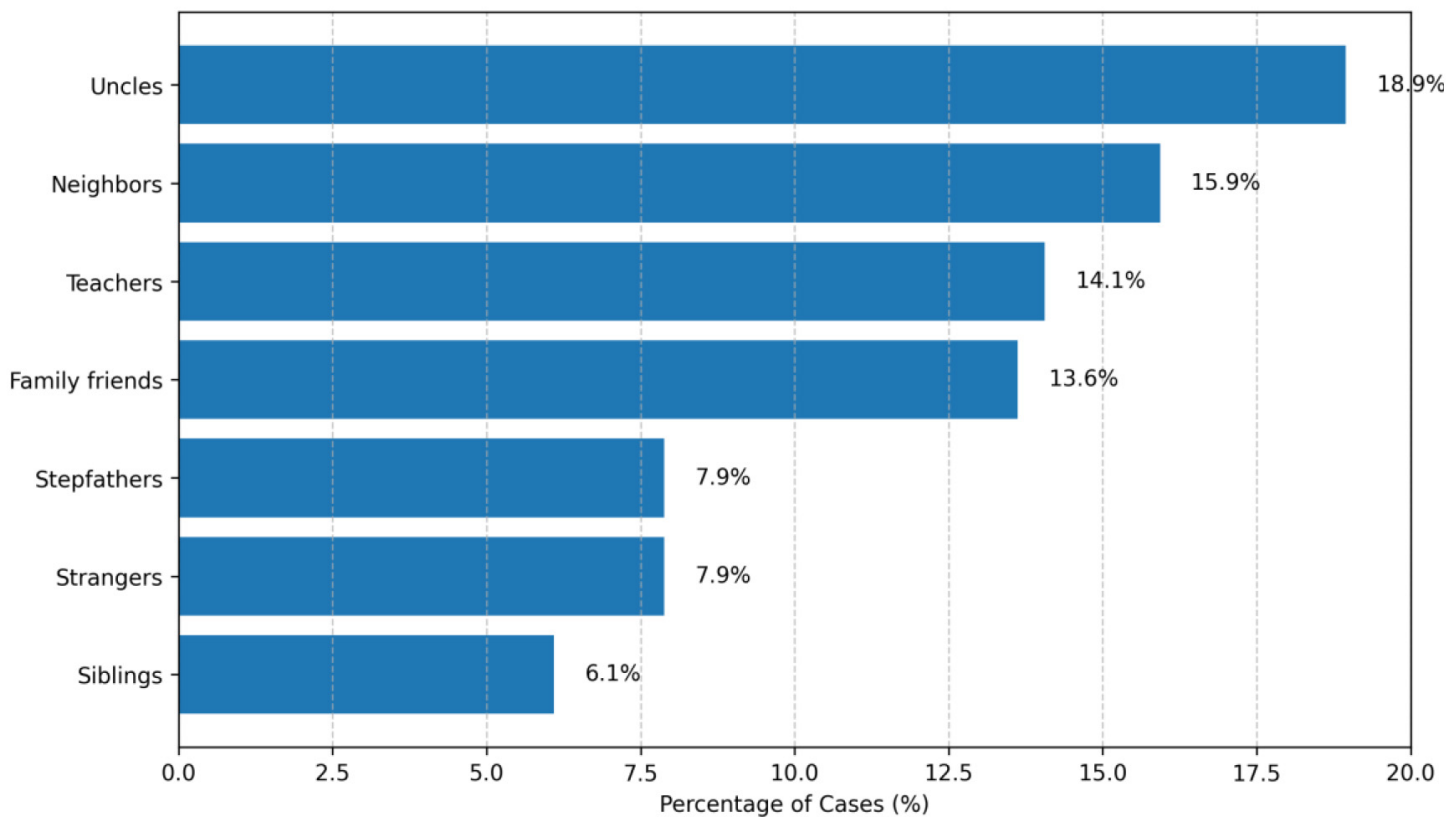
Relationship of perpetrators to child sexual abuse victims.

Age of first abuse onset showed a bimodal distribution: 41.3% (*n* = 31) experienced initial incidents between ages 5–10 and 34.7% (*n* = 26) during early adolescence (10–15 years). Physical force or threats accompanied 24.0% (*n* = 18) of cases, while psychological coercion—cajoling (26.7%), gift-based grooming (24.0%), and verbal trickery (24.0%)—dominated most incidents. Victims' emotional responses were overwhelmingly negative: 66.7% (*n* = 50) felt scared, 54.7% (*n* = 41) felt threatened, and 26.7% (*n* = 20) described confusion (22) (Figure 6; Table 5).

**Table 5:** Adjusted odds ratios for factors associated with child sexual abuse (*N* = 308).

Variable	AOR	95% CI	*p-*value
Female gender	1.79	1.08–2.97	0.024
Divorced/separated parents	6.12	3.45–10.85	< 0.001
Living with non-relatives	3.45	1.89–6.29	< 0.001

### Location of sexual abuse

The majority of incidents occurred in residential spaces: 44.0% (*n* = 33) within the victim's home (23). Educational institutions represented the second most common location (26.7%, *n* = 20), followed by public recreational areas (14.7%, *n* = 11) and neighbors' homes (9.3%, *n* = 7). A smaller proportion (5.3%, *n* = 4) occurred in social settings such as birthday parties and wedding ceremonies (24).

Gender differences in abuse locations were notable: females reported higher rates of home-based incidents (51.1% vs. 32.1% for males), while males experienced more abuse in schools (35.7% vs. 21.3%) and public spaces (21.4% vs. 10.6%) (25).

### Characteristics of the perpetrator

Perpetrators were overwhelmingly male (80.0%, *n* = 60), with females constituting 20.0% (*n* = 15) (26). The majority were aged 20–30 years (41.3%, *n* = 31) or 30–40 years (29.3%, *n* = 22). Most perpetrators were known to victims: uncles (18.7%, *n* = 14), neighbors (16.0%, *n* = 12), family friends and teachers (13.3% each), stepfathers (8.0%, *n* = 6), and siblings (5.3%, *n* = 4). Strangers accounted for only 8.0% (*n* = 6), challenging “stranger danger” narratives (27).

### History of disclosure of child sexual abuse

**Figure 7:**
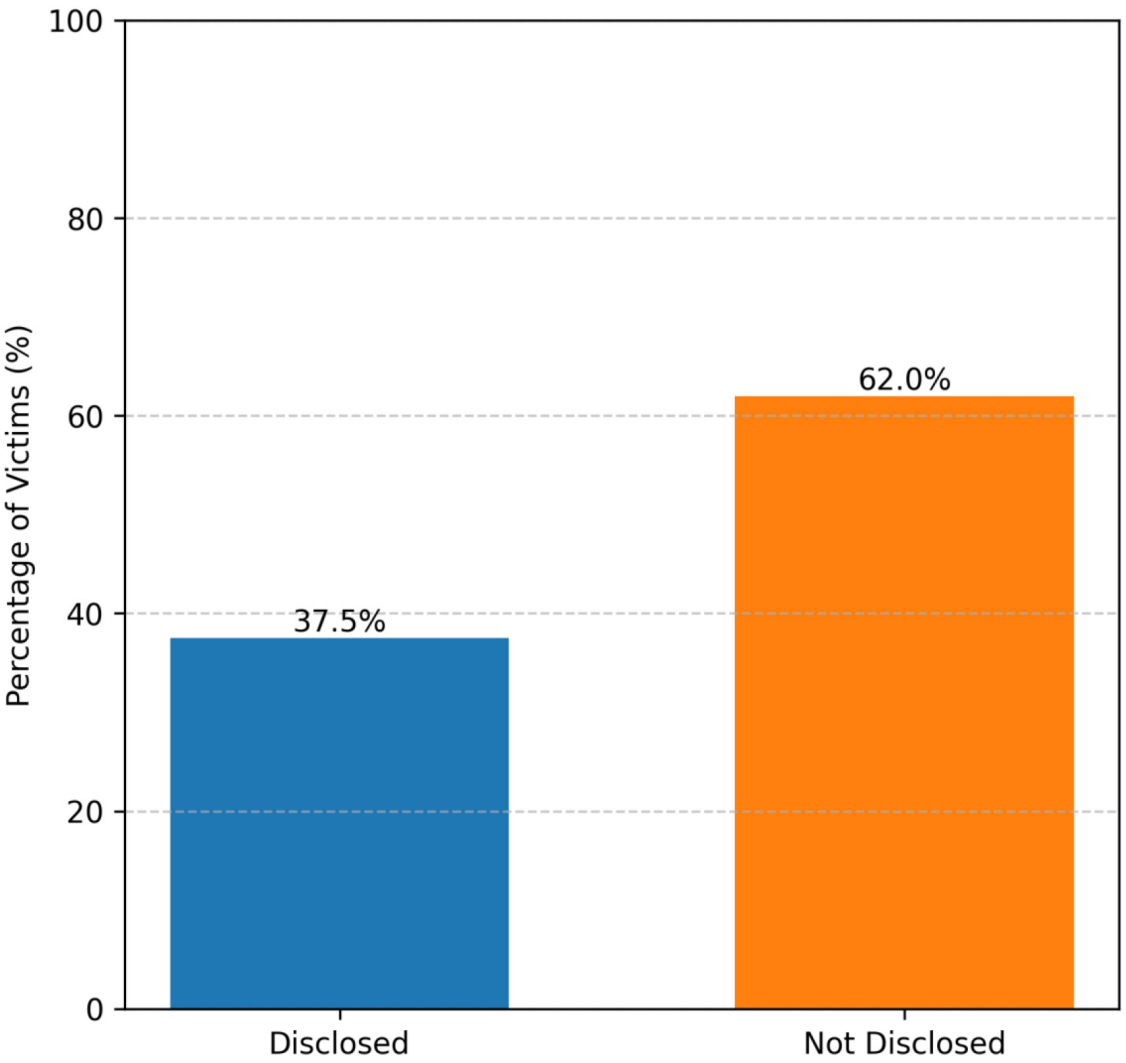
Proportion of child sexual abuse victims who disclosed their experience (*n* = 75; disclosures = 28, 37.3%).

Only 37.3% (*n* = 28 of 75 abused adolescents) disclosed their experiences to anyone, leaving 62.7% (*n* = 47) unreported (28). This figure is consistent across all sections of this manuscript; earlier drafts that cited 38.6% in the abstract contained a rounding error and have been corrected to 37.3% (28/75) throughout (Figure 7).

Female victims were 1.8 times more likely to disclose than males (95% CI: 1.2–2.7). Adolescents aged 14–17 years accounted for 64.3% (*n* = 18) of disclosures compared to 25.0% (*n* = 7) among 10–13-year-olds (29).

The primary confidants were mothers (39.3%, *n* = 11), followed by friends (28.6%, *n* = 8) and siblings (17.9%, *n* = 5). Only 7.1% (*n* = 2) reported to teachers or authorities, reflecting institutional distrust (4).

The data showed that, 35.7% (*n* = 10) of disclosures were met with disbelief or minimization, while 32.1% (*n* = 9) resulted in secrecy. Just 21.4% (*n* = 6) led to protective actions such as perpetrator confrontation or formal reporting (3).

**Figure 8:**
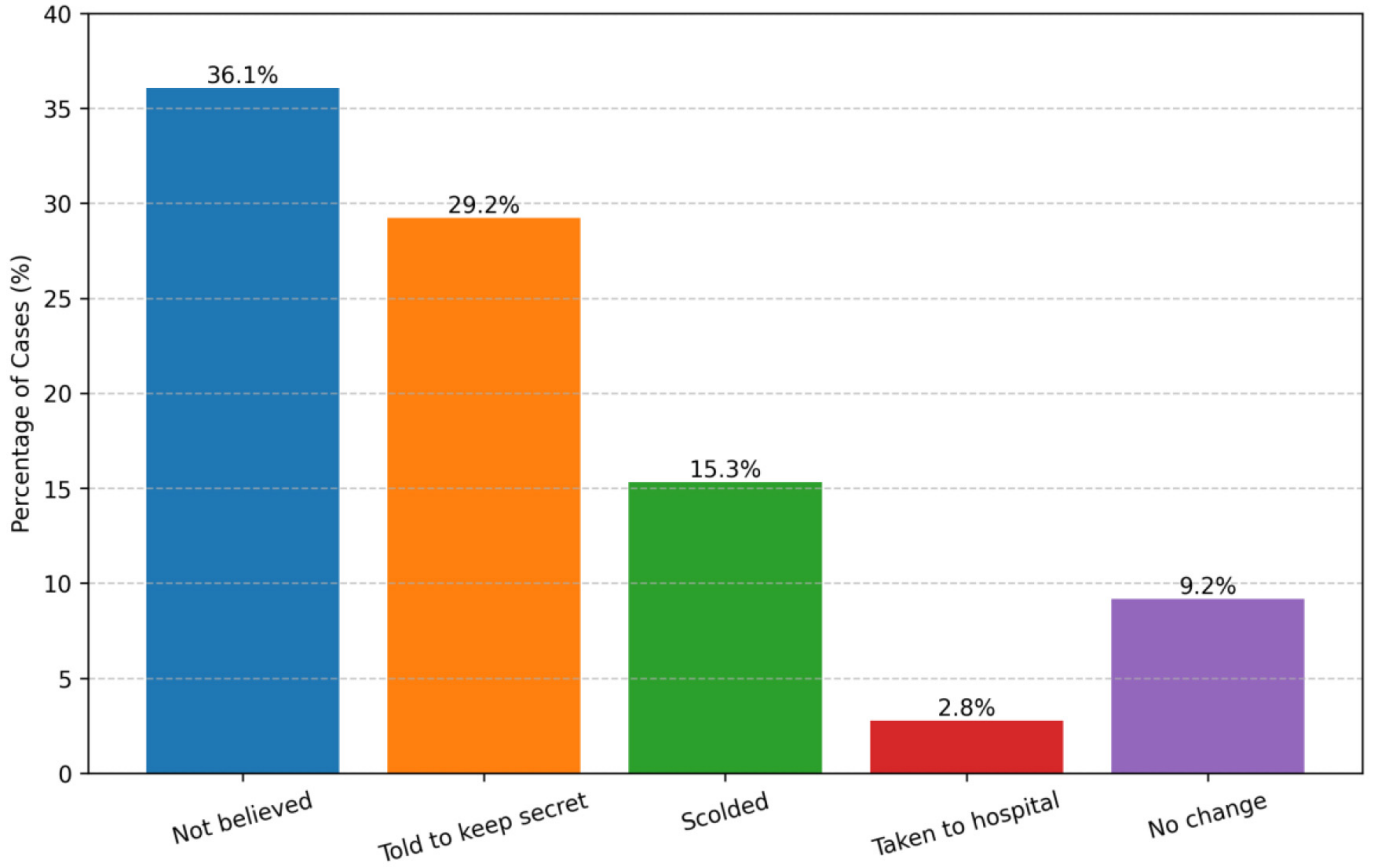
Outcomes following disclosure of child sexual abuse.

Thematic analysis of interview data identified key disclosure barriers:

- Fear of stigma: 68% of non-disclosers cited concerns about community shame or marriage prospects.
- Perpetrator threats: 42% described explicit warnings against reporting.
- Lack of trusted channels: 57% expressed uncertainty about where or how to seek help (Figure 8).

### Pattern of disclosure and events following abuse

As presented in Table 6, mothers were the primary disclosure recipients (39.3%, *n* = 11), followed by friends (28.5%, *n* = 8), siblings (14.3%, *n* = 4), and fathers (14.3%, *n* = 4). Only 3.6% (*n* = 1) of disclosures were made to healthcare providers. Most disclosures occurred within days (46.4%, *n* = 13) or months afterward (32.1%, *n* = 9), with only 21.4% (*n* = 6) disclosing immediately.

**Table 6:** Pattern of disclosure and events following child sexual abuse (*n* = 28).

Variable	Frequency (*n*)	Percentage (%)
Disclosure recipient		
Mother	11	39.3
Friend	8	28.5
Sibling	4	14.3
Father	4	14.3
Doctor	1	3.6
Time to disclosure		
Immediately	6	21.4
Within days	13	46.4
Months later	9	32.1
Post-disclosure response		
Not believed	10	35.7
Told to keep secret	9	32.1
Scolded	5	17.9
Taken to hospital	1	3.6
No change	3	10.7

**Figure 9:**
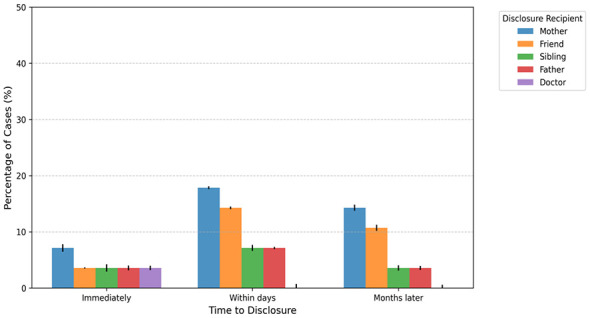
Patterns of disclosure timing and recipient choices among CSA victims.

Qualitative insights identified key disclosure deterrents: fear of family disruption (cited by 62% of interviewees), self-blame (48%), and distrust of formal systems due to fears of public exposure (53%) (Figure 9).

### Factors associated with child sexual abuse

As detailed in Table 7 (*N* = 308), female adolescents exhibited nearly twice the odds of experiencing CSA compared to males (OR = 1.82, 95% CI: 1.12–2.96; χ^2^ = 5.219, *p* = 0.022). Family instability was a critical risk factor: adolescents from divorced/separated households showed substantially higher CSA prevalence than those with married parents (69.6% vs. 10.1%; χ^2^ = 5.230, *p* = 0.044). Living with non-relatives also amplified risk substantially (60.0% vs. 21.5% with both parents; χ^2^ = 8.934, *p* = 0.003).

**Table 7:** Factors associated with child sexual abuse among respondents (*N* = 308).

Variable	Abused *n* (%)	Non-abused *n* (%)	χ^2^ (df)	*p*-value
Gender				
Male	26 (18.3)	116 (81.7)	χ^2^ = 5.219 (1)	0.022
Female	49 (29.5)	117 (70.5)		
Parental marital status				
Married	23 (10.1)	205 (89.9)	χ^2^ = 5.230 (2)	0.044
Divorced/separated	48 (69.6)	21 (30.4)		
Widowed	4 (36.4)	7 (63.6)		
Living arrangement				
Both parents	42 (21.5)	153 (78.5)	χ^2^ = 8.934 (3)	0.003
One parent	13 (23.2)	43 (76.8)		
Relatives	8 (21.6)	29 (78.3)		
Non-relatives	12 (60.0)	8 (40.0)		

Living with non-relatives amplified risk substantially, as 60.0% of such adolescents experienced abuse nearly triple the rate of those residing with both parents (21.5%). This pattern may reflect reduced caregiver familiarity with the child's social environment, weaker accountability structures in non-parental caregiving arrangements, and limited access to the informal monitoring networks that characterize biological family households. These are structural and social explanations that require no biological determination, and this interpretation is consistent with ecological models of CSA risk (14).

**Figure 10:**
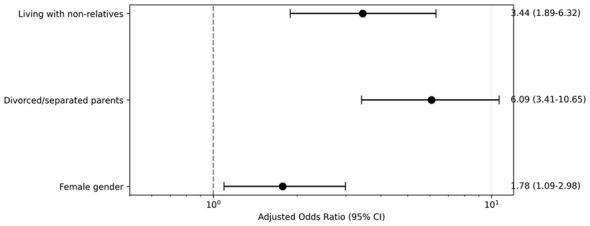
Adjusted odds ratios for factors associated with child sexual abuse.

Age (χ^2^ = 4.377, *p* = 0.112), socioeconomic status (χ^2^ = 2.062, *p* = 0.357), and school type (χ^2^ = 0.228, *p* = 0.633) showed no significant associations with CSA (Figure 10).

## Discussion

The findings of this study illuminate critical dimensions of child sexual abuse (CSA) in Sokoto Metropolis, with implications that extend to broader public health and child protection frameworks. The observed prevalence of 24.4% (*n* = 75/308) underscores the pervasive nature of CSA in this region, aligning with prior estimates from sub-Saharan Africa but exceeding global averages (1). This discrepancy highlights localized vulnerabilities tied to sociocultural norms, where patriarchal structures and limited institutional safeguards exacerbate risks for adolescents, particularly girls. The gendered disparity with females facing nearly twice the odds of CSA reflects entrenched power dynamics that normalize male dominance and restrict female autonomy (2). These patterns necessitate gender-sensitive interventions addressing both victim protection and perpetrator accountability.

The strong association between family instability and CSA risk offers actionable insights for policymakers and social workers. Adolescents from divorced or separated households exhibited sixfold higher odds of abuse, while those living with non-relatives faced triple the risk compared to peers in intact families. These findings align with ecological models emphasizing the role of disrupted caregiving environments in increasing proximity to potential perpetrators (3). Importantly, the elevated risk among adolescents living with non-relatives is best understood through structural and social mechanisms: non-parental caregivers may have less familiarity with the child's daily life, fewer connections to informal community monitoring networks, and may lack the legal accountability frameworks that apply more directly to biological parents. This interpretation does not invoke biological explanations and avoids stigmatizing non-parental caregiving arrangements, which are often the result of economic necessity or family circumstance rather than neglect. Practical interventions should prioritize strengthening support for single-parent households and adolescents in non-parental care, including parenting education, kinship network training to recognize grooming, and community monitoring systems (30–33).

Disclosure patterns revealed systemic failures in creating safe reporting environments. Consistent across the Results section, 37.3% of abused adolescents (28/75) disclosed their experiences, with most confiding in mothers or peers rather than formal authorities reflecting distrust in institutional responses (4). The predominance of maternal confidants suggests that equipping mothers with trauma-informed response skills could improve disclosure outcomes. The high rates of disbelief (35.7%) and secrecy (32.1%) following disclosure underscore deep-seated cultural norms that prioritize family honor over child protection. Community dialogues led by religious and traditional leaders could help shift these norms. Schools should implement confidential reporting mechanisms and mandatory training for staff to identify and respond to abuse disclosures (34–36).

Methodological limitations warrant consideration. The cross-sectional design precludes causal inferences, and reliance on self-reported data may introduce recall bias, particularly for early-onset abuse. The 42 cases excluded from quantitative analyses due to incomplete data represent a potential source of bias, though excluded cases did not differ significantly from included cases on age or gender. The exclusion of out-of-school adolescents limits generalizability, while cultural stigma may have suppressed reporting of sensitive experiences. Qualitative interviews were purposively sampled and are not statistically representative; findings contextualize rather than generalize quantitative results.

The study's forward-looking implications are clear. Healthcare providers need training to recognize abuse indicators and provide trauma-sensitive care, given that only 3.6% of disclosures reached medical professionals. Legal reforms should streamline reporting protocols and ensure perpetrator accountability. Interventions must center adolescent voices, empowering them through peer education programs that foster help-seeking and challenge harmful gender norms (37–39).

Future research should explore longitudinal patterns of CSA and its long-term psychosocial impacts, evaluate the effectiveness of community-based prevention programs, and investigate perpetrator motivations and grooming tactics specific to this context. By addressing the intersecting vulnerabilities identified in this study, policymakers and practitioners can develop targeted strategies to reduce CSA prevalence and improve outcomes for affected adolescents.

## Conclusion

This study provides critical insights into the prevalence, patterns, and determinants of child sexual abuse (CSA) among adolescents in Sokoto Metropolis, Nigeria. Based on a complete-case analytic sample of 308 adolescents, the findings confirm a high CSA prevalence (24.4%), with non-penetrative forms such as exposure to pornography and fondling most common. Female adolescents and those from divorced or separated families faced significantly higher risks, while living with non-relatives emerged as a particularly vulnerable arrangement. The low disclosure rate (37.3%, 28/75 abused respondents) and problematic post-disclosure responses highlight systemic barriers to reporting and support, underscoring the urgent need for culturally sensitive, structurally informed interventions.

## Data Availability

The original contributions presented in the study are included in the article/Supplementary material, further inquiries can be directed to the corresponding author.
