# Supplementary material for: Disclosure pathways and correlates of child sexual abuse among secondary school adolescents in Sokoto metropolis, Nigeria
Source: Front Public Health. 2026 Jul 28;14:1805885. doi: 10.3389/fpubh.2026.1805885 (PMC13457455; doi:10.3389/fpubh.2026.1805885)
Supplement: Supplementary file 1 [file Data_Sheet_1.pdf]

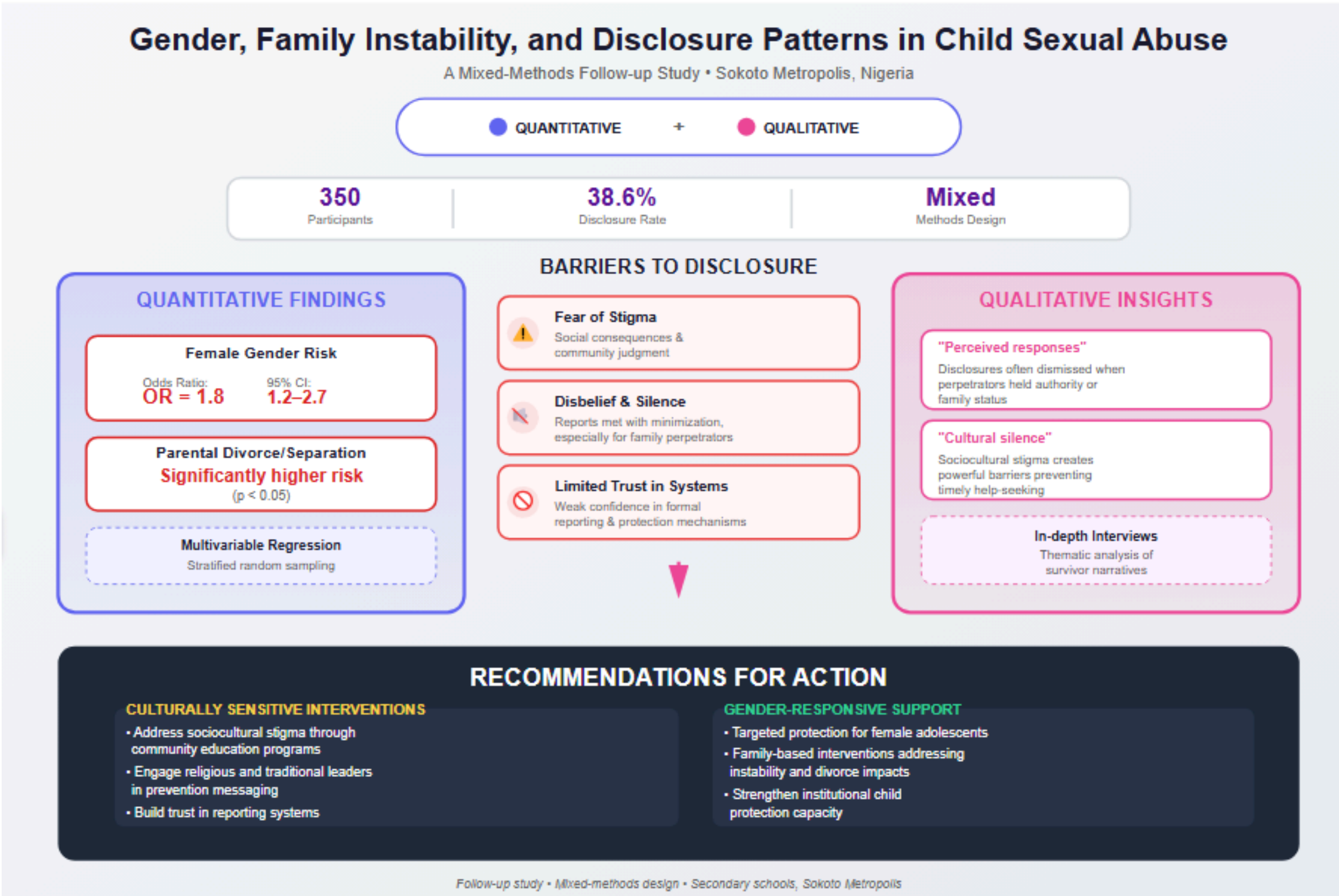
Gender, Family Instability, and Disclosure Patterns in Child Sexual Abuse
A Mixed-Methods Follow-up Study • Sokoto Metropolis, Nigeria
QUANTITATIVE + QUALITATIVE
350
Participants
38.6%
Disclosure Rate
Mixed
Methods Design
QUANTITATIVE FINDINGS
Female Gender Risk
Odds Ratio:
OR = 1.8
95% CI:
1.2–2.7
Parental Divorce/Separation
Significantly higher risk
(p < 0.05)
Multivariable Regression
Stratified random sampling
BARRIERS TO DISCLOSURE
Fear of Stigma
Social consequences & community judgment
Disbelief & Silence
Reports met with minimization, especially for family perpetrators
Limited Trust in Systems
Weak confidence in formal reporting & protection mechanisms
QUALITATIVE INSIGHTS
"Perceived responses"
Disclosures often dismissed when perpetrators held authority or family status
"Cultural silence"
Sociocultural stigma creates powerful barriers preventing timely help-seeking
In-depth Interviews
Thematic analysis of survivor narratives
RECOMMENDATIONS FOR ACTION
CULTURALLY SENSITIVE INTERVENTIONS
• Address sociocultural stigma through community education programs
• Engage religious and traditional leaders in prevention messaging
• Build trust in reporting systems
GENDER-RESPONSIVE SUPPORT
• Targeted protection for female adolescents
• Family-based interventions addressing instability and divorce impacts
• Strengthen institutional child protection capacity
Follow-up study • Mixed-methods design • Secondary schools, Sokoto Metropolis
